# Motivational determinants of frontline health extension workers in East Hararge Zone, Ethiopia: implications for primary health care systems in low-resource settings

**DOI:** 10.3389/fpubh.2025.1678937

**Published:** 2025-11-19

**Authors:** Bereket Yonas Madebo, Debela Tezera Simagn, Yonas Meheretu

**Affiliations:** 1 School of Postgraduate Studies, Department of Management, Rift Valley University, Harar, Ethiopia; 2 Department of Wildlife, Fish and Environmental Studies, Swedish University of Agricultural Sciences, Umeå, Sweden

**Keywords:** primary health care, health extension workers, workforce motivation, intrinsic and extrinsic factors, community health systems, Ethiopia

## Abstract

**Background:**

Health worker motivation affects service quality, retention, and the performance of primary health systems, especially in low-income settings. In Ethiopia, Health Extension Workers (HEWs) form the backbone of the rural health system, yet limited empirical evidence exists on the factors influencing their motivation across diverse regional contexts.

**Methods:**

A survey was conducted with 314 HEWs from six Woreda (Districts) and one town administration in the Oromia Region of Ethiopia, between July 1 and August 14, 2024. Motivation was operationally defined as the capacity of HEWs to initiate, direct, and maintain goal-oriented working behaviors. Descriptive statistics and logistic regression analyses were performed to identify significant associations between key socio-demographic and institutional factors with motivation, with results expressed as adjusted odds ratios (AOR).

**Results:**

Overall level of motivation among the HEWs was 74.8%. Both intrinsic (socio-demographic) and extrinsic (institutional) factors were significantly associated with motivation. Being married was strongly associated with motivation compared to being single (AOR = 222.93; CI:95%; *p* < 0.05), as it was having >15 years of service (AOR = 5.79; CI:95%; *p* < 0.05) compared to >1 year of service. Institutional factors: satisfaction with performance related incentives (AOR = 2.46; CI: 95%; *p* < 0.05), knowledge and skill sharing with co-workers (AOR: 3.52; CI:95%; *p* < 0.05) and decision-making autonomy (AOR = 3.114; CI:95%; *p* < 0.05) were strong predictors of higher motivation. In contrast, inadequate implementation of the career development system (AOR = 0.510; CL:95%; *p* < 0.05), routine supervision and feedback (AOR = 0.503; CI: 95%; *p* < 0.05), and access to on-the-job or off-the-job training (AOR: 0.44; CI:95%; *p* < 0.05) were linked with lower motivation.

**Conclusion:**

We found a relatively high motivation among HEWs in East Hararge compared to other regions in Ethiopia and Africa. Both intrinsic and extrinsic motivators identified play a key role in shaping the motivation levels of HEWs. To enhance worker motivation and productivity in the primary health care system, policymakers and health sector authorities need to implement targeted and evidence-based strategies, including structured career development pathways, performance-based incentives, strong collegial knowledge and skill sharing schemes, and decision-making autonomy.

## Introduction

The term “motivation” originates from the Latin word “movere,” meaning “to move.” In psychology, motivation is the process that initiates, guides, and sustains goal-directed behavior. In organizational settings, it embodies both intrinsic and extrinsic forces that drive individuals to act toward achieving specific objectives (1). Over the years, foundational theories such as Maslow’s Hierarchy of Needs, Herzberg’s Two-Factor Theory, and Deci and Ryan’s Self-Determination Theory have provided critical frameworks for understanding motivation, particularly the interplay between intrinsic and extrinsic factors (2–6).

Maslow’s theory suggests that human motivation is driven by the fulfilment of hierarchical needs, beginning with basic physiological needs and progressing through safety, social belonging, esteem, and culminating in self-actualization (2). These hierarchal needs are often represented as a pyramid, with foundational needs (e.g., food, water) at the base, and higher-order psychological and self-fulfilment needs at its apex. For instance, in workplace settings, ensuring employee safety (e.g., secure job conditions) and belongingness (e.g., collegial relationships) can significantly enhance employee motivation. Recent studies have extended Maslow’s framework to modern organizational contexts. For example, Gopalan et al. (7) demonstrated that addressing employees’ psychological needs, such as recognition and professional growth, contributes to increased job satisfaction and enhanced productivity.

Herzberg’s Two-Factor Theory categorizes workplace factors into two groups: hygiene factors and motivators (3). Hygiene factors, such as salary, job security, and working conditions, prevent dissatisfaction but do not necessarily enhance genuine motivation. In contrast, motivators, such as recognition, meaningful work, and opportunities for personal growth, drive higher levels of job satisfaction and intrinsic motivation. For example, Lu et al. (8) in the nursing profession found that while working conditions reduced turnover, motivators such as professional development opportunities and supervisory support were key to sustaining high levels of motivation and job satisfaction.

Complementing this framework, Deci and Ryan’s Self-Determination Theory (SDT) emphasizes that intrinsic motivation arises when three fundamental psychological needs are met: autonomy, competence, and relatedness (4). According to SDT, individuals are most motivated when they perceive a sense of control over their actions (autonomy), feel effective in their tasks (competence), and connected to others (relatedness). In practice, promoting autonomy through flexible work arrangements has been shown to enhance employee satisfaction and performance. For instance, Olafsen et al. (9) showed that employees in organizations that encouraged autonomy and provided opportunities for competence development show optimal levels of engagement and intrinsic motivation.

In the context of Sub-Saharan Africa, health workers’ motivation is a key determinant of healthcare delivery quality and consistency (10). Despite notable progress in improving access to healthcare across the region, maintaining high levels of motivation and engagement among health personnel remains a persistent challenge, particularly in settings characterized by limited resources and systemic constraints (11). Empirical studies underscore the significance of financial incentives, structured career development pathways, and supportive supervision in enhancing health worker motivation (12, 13). For instance, a study in Tanzania indicated that dissatisfaction with low remuneration and inadequate working conditions undermines the motivation of primary healthcare workers (14).

In Ethiopia, studies on health worker motivation have primarily centered on hospital-based professionals, consistently highlighting the role of financial incentives, career advancement, and supportive work environments in enhancing motivation (15). In contrast, limited attention has been given to the motivation of health extension workers (HEWs), despite they constitute the backbone of the country’s Health Extension Program (HEP). The HEP is a nationally implemented initiative designed to provide primary healthcare services to rural populations, with each health post typically serving between 3,000 and 5,000 individuals within a Kebele (the smallest administrative unit in Ethiopia) (16). Employed by the Ethiopian Ministry of Health, HEWs are predominantly women who have completed at least a 10th-grade education, followed by a one-year intensive training program. This training equips them with the competencies required to deliver 16 standardized health service packages that cover maternal and child health, hygiene and sanitation, disease prevention, and health education (17, 18). Given their pivotal role in advancing universal health coverage at the grassroots level, understanding the motivational drivers and barriers affecting HEWs is critical, yet remains under-explored in the current literature. Recent reviews [e.g., (19)]. also show that HEW studies overwhelmingly focus on service delivery, capacity building, or efficiency, with motivation typically treated as a secondary issue rather than a central theme.

The motivation of HEWs is critical for the success and sustainability of Ethiopia’s primary healthcare strategy. However, comparative analyses across the country’s diverse regions show substantial disparities in HEWs job satisfaction and motivation and emphasize the need for localized assessments that account for regional contexts (12). For instance, reported job satisfaction rates among HEWs in Hadiya, Sidama, and West Hararge Zones (regions) range from 16.6 to 52.7% (13, 20, 21). In West Shewa Zone, studies have demonstrated that health workers who received financial incentives exhibited significantly higher levels of motivation (22). Conversely, data from Gedeo Zone reported critically low motivation levels (19.5%) among public health personnel, attributing this decline to factors such as resource shortages, insufficient remuneration, lack of supervisory feedback, limited training opportunities, substandard working environments, and excessive workloads (23).

To contribute to the growing body of evidence on health worker motivation in resource-constrained settings, and to address the gap in region-specific data within Ethiopia, this study focuses on the East Hararge Zone, Eastern Ethiopia. Specifically, we investigated the determinants of motivation among HEWs operating in this region. We provide region-specific insights to support policy decisions and HEP authorities in designing targeted interventions that enhance HEW motivation, improve primary healthcare delivery, and ultimately contribute to better health outcomes in the region.

## Methods

### Study area and population

**Figure 1:**
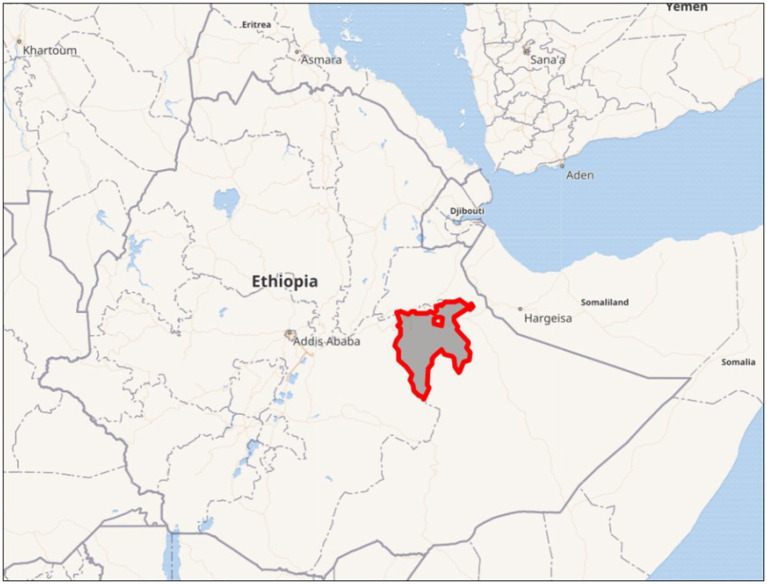
Map showing the geographic location of East Hararge Zone (shaded in grey and outlines in red) in Easter Ethiopia.

The study was conducted in the East Hararge Zone, located in the Oromia National Regional State of Ethiopia, with Harar as its capital town located ca. 525 km east of Addis Ababa (Figure 1). The zone comprises 21 Woredas (district-level administrative units) and two town administrations (local government entities responsible for administrating urban towns). Health extension workers (HEWs) in these area are assigned to health posts and community health centers, which are characterized by a lack of inpatient facilities and are primarily tasked with delivering essential community-based health services. The study targeted all HEWs actively working in six randomly selected Woredas and one town administration using the lottery method. Eligibility criteria included: (1) formal deployment by the respective Woreda or town administration, and (2) active service at a health post within a Kebele.

### Sample size determination

A multistage random sampling procedure was employed. First, 30% of the Woredas and town administration within the zone were selected randomly. Next, health posts within the chosen administrative units were randomly sampled. In the final stage, individual HEWs were proportionally allocated and selected from the sampled health posts based on their staffing levels.

The required sample size was calculated using a single population proportion formula, assuming an estimated motivation rate of 61% among HEWs reported in previous studies from Amhara, Oromia, Southern, and Tigray regions (24). The parameters applied included a 95% confidence interval, a 5% margin of error, and a design effect of 1.0, resulting in a minimum sample size of 365 participants. In practice, 314 HEWs were surveyed due to logistical limitations and the exclusion of incomplete responses.

The final sample was distributed across districts as follows: Kombolcha (*n* = 41), Haramaya (*n* = 36), Babile (*n* = 49), Kersa (*n* = 65), Meta (*n* = 56), Goro Gutu (*n* = 57), and Babile Town (*n* = 10). The study was conducted between July 1st and August 14th, 2024.

### Data collection

Data were collected using structured, pretested questionnaire, designed to gather quantitative and qualitative information on HEWs motivation, work engagement, and related factors. A team comprising five trained data collectors and two supervisors conducted a face-to-face interview after completing a half-day training session on standardized data collection procedures and ethical considerations. Questionnaires were administered in Afan Oromo, the local language, following ethical approval and permission from relevant Woreda health offices. The data collectors ensured participant confidentiality and obtained verbal informed consent prior to initiating the interviews.

### Data management and analysis

Data were checked for completeness and consistency, coded, and entered into a customized Microsoft Excel template before being exported to IBM SPSS version 27.0.1 for analysis. Motivation was operationally defined as the capacity of HEWs to initiate, direct, and maintain goal-oriented working behaviors. Descriptive statistics were used to summarize socio-demographic characteristics and key outcome measures. Bivariate and multivariate binary logistic regression analyses were performed to evaluate associations between motivation (outcome) and independent (intrinsic and extrinsic) variables. In the bivariate analysis, crude odds ratios (CORs) were calculated for each explanatory variable. Variables with *p*-values < 0.25 in the bivariate analysis were considered for multivariate analysis. Prior to conducting the multivariate analysis, multicollinearity was assessed using Variance Inflation Factor (VIF) thresholds. Final models reported adjusted odds ratios (AORs) with 95% confidence intervals, identifying factors independently associated with HEWs’ motivation.

### Ethical considerations

Ethical clearance was obtained from Rift Valley University, Harar Campus, and subsequently presented to the East Hararge Zone health office for approval. The zonal health office issued approval letters to the six Woredas and one town administration. Following this, the respective Woreda and Town Administration health offices granted further authorization to each health post allowing data collection. Prior to participation, all respondents were carefully informed about the objectives and procedures of the study. Written informed consent was obtained from each participant, with the consent from prepared in Afan Oromo, the local language. Participation was entirely voluntary, and participants were informed about their rights to withdraw at any point without any consequence. The study adhered to the core ethical principles of autonomy, respecting human dignity, individual rights, and freedom. Throughout the data collection process, the privacy and confidentiality of respondent information were upheld by maintaining anonymity.

## Results

### Socio-demographic characteristics

A total of 314 HEWs from six Woredas and one town administration participated in this study, achieving a response rate of 86% relative to the calculated sample size. The distribution of respondents across locations showed that the highest proportion were from Kersa, 65 (20.7%), followed by Goro Gutu, 57 (18.2%), while the fewest were from Babile Town, 10 (3.2%) (Table 1). In terms of age distribution, nearly half of the respondents, 154 (49.0%), were in the age group 28–37 years, followed by 126 (40.2%) in the 18–27 age group. Regarding marital status, the majority were married, 244 (77.7%), while a few were widowed, 8 (2.6%). Most respondents held a diploma, 227 (72.3%), while only 5 (1.6%) had an educational level between Grade 10 and 12. Religious affiliation was predominantly Muslim, 255 (81.2%), followed by Orthodox Christian, 52 (16.5%). With respect to work experience, 84 (26.8%) had served between 11 and 15 years, 83 (26.4%) between 6 and 10 years, and 67 (21.3%) had 1–5 years of service. A small group, 15 (4.8%), reported less than 1 year of service. All respondents were female.

**Table 1:** Socio-demographic characteristics of the health extension workers (HEWs) (*n* = 314) interviewed in the East Hararge Zone, August 2024.

Variables	Characteristics	Count	Percent
Age	18–27	126	40.2
	28–37	154	49
	>38	34	10.8
Address	Kombolcha	41	13.1
	Haramaya	36	11.4
	Babile	49	15.6
	Kersa	65	20.7
	Meta	56	17.8
	Goro Gutu	57	18.2
	Babile Town	10	3.2
Marital status	Single	38	12.1
	Married	244	77.7
	Divorced	24	7.6
	Widowed	8	2.6
Educational status	Grade 10–12	5	1.6
	Certificate	70	22.3
	Diploma	227	72.3
	Degree	12	3.8
Religion	Muslim	255	81.2
	Orthodox	52	16.5
	Protestant	4	1.3
	Catholic	3	1
Service year	<1 year	15	4.8
	1–5 year	67	21.3
	6–10 year	83	26.4
	11–15 year	84	26.8
	>15 year	65	20.7

### Motivation and motivational factors

**Figure 2:**
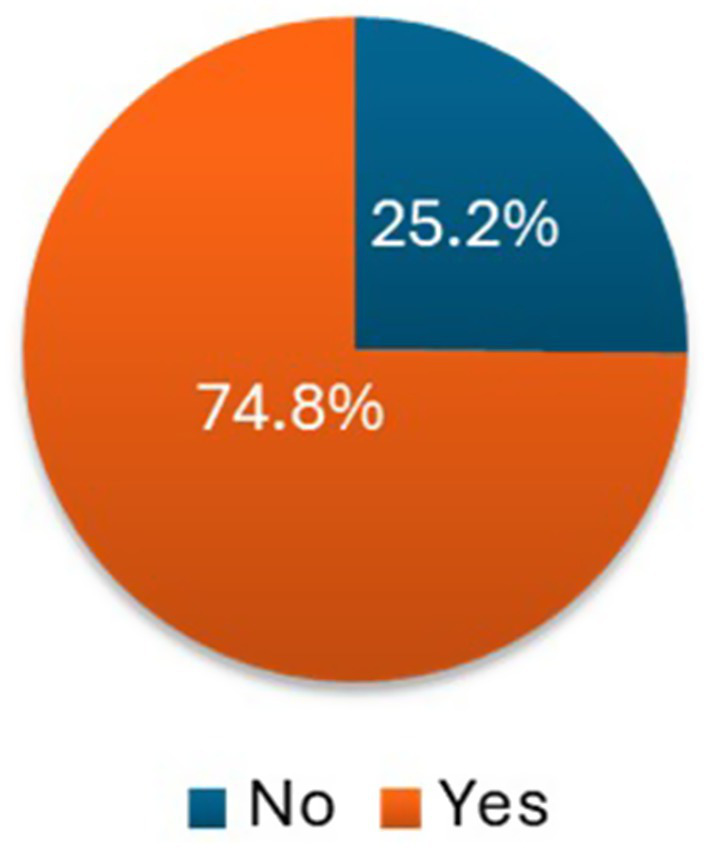
The overall motivation level of the HEWs.

**Figure 3:**
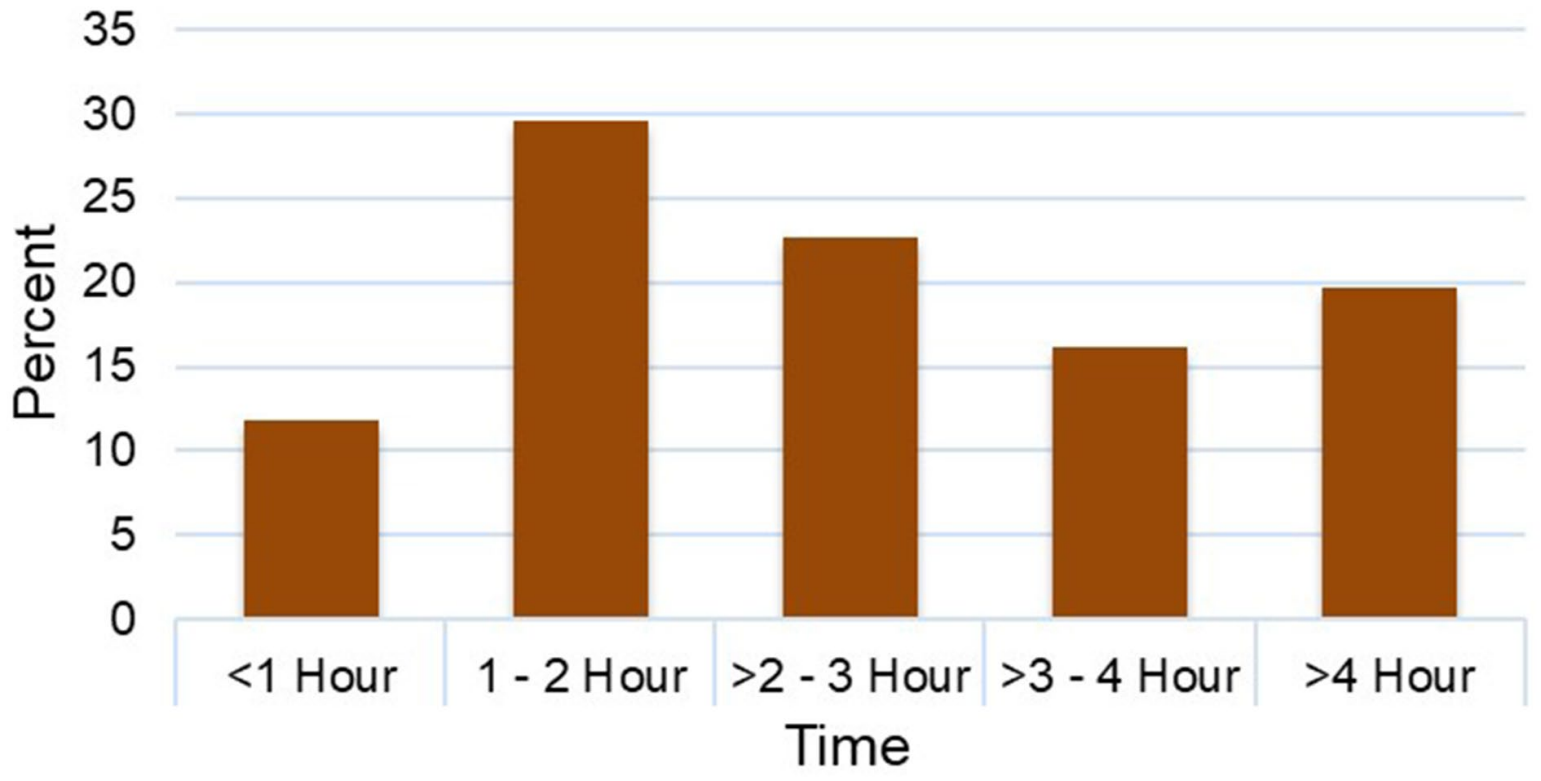
Daily commuting time among HEWs from their residence to their assigned health posts.

**Figure 4:**
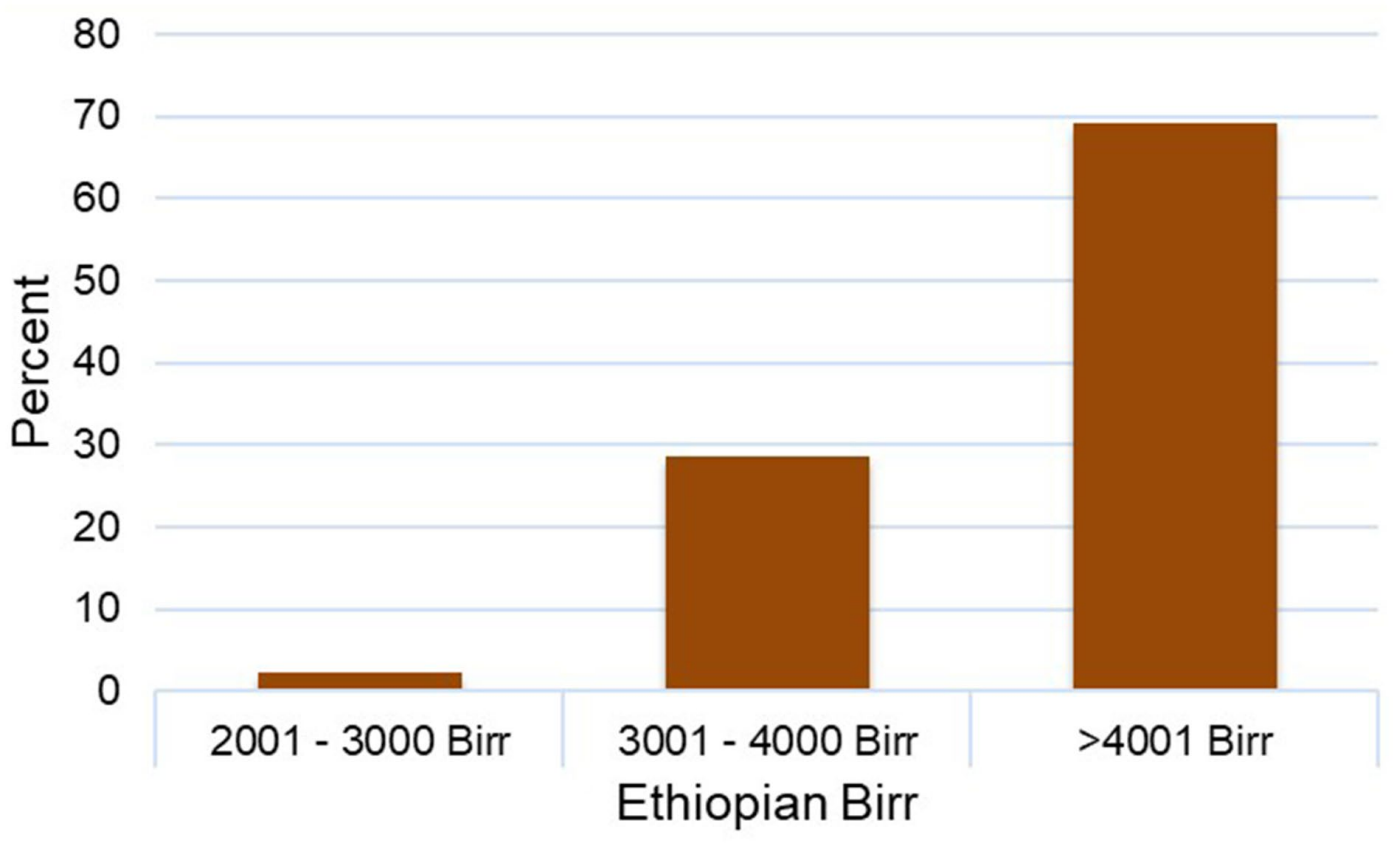
Monthly gross salary distribution of HEWs in Ethiopian Birr.

The overall motivation level of the HEWs reported was 74.8% (Figure 2). Analysis of motivational factors showed that the majority of the HEWs surveyed (68.4%) worked at health posts located within 1 to 4 hours walking distance from their town of residence (Figure 3). Among these, 29.6% reported walking between 1 to 2 hours daily, while nearly 20% reported walking for over 4 hours each working day to reach their health posts. With respect to salary distribution, most HEWs (69.1%) reported earning a monthly gross salary of slightly greater 4,000 Ethiopian Birr (ETB) (ca. USD 50, based on the average exchange rate at the time of data collection) (Figure 4). An additional 29.4% reported earning between 3,001–4,000 ETB, while a small fraction (1.5%) reported earning a salary range of 2,001–3,000 ETB.

Additional factors affecting the motivation of HEWs surveyed were presented in Table 2. Community recognition emerged as a major motivator, with 65.6% of HEWs strongly agreeing that the communities they serve genuinely need their services (Table 2). This sense of necessity is further validated by 65.9% of the HEWs strongly agreeing that improvements in community health outcomes reflect appreciation for their work and robust community support. Career development appears less structured, with only 26.4% strongly agreeing that a career support system exists within the Health Office, while 25.5% remained neutral. Despite limited structural support, 54.5% strongly agreed that career development opportunities are crucial to enhancing their performance. About 33% of participants strongly agreed that they receive regular and constructive feedback from their supervisors, while a greater proportion, 44.9%, strongly agreed that supervisory feedback effectively motivates them to improve their performance. Training needs were strongly acknowledged by HEWs, where slightly higher than half (54.8%) strongly agreed that their job requires regular on-the-job and off-the-job training, and 65.9% emphasized the importance of sharing knowledge and skills with co-workers to enhance collective performance. In terms of incentives and decision-making (autonomy), 41.4% expressed strong satisfaction with their current opportunities for promotion, transfer, and professional advancement, while 55.1% strongly agreed they have the autonomy to make independent decisions in their work.

**Table 2:** Factors influencing motivation of HEWs in East Hararge Zone.

Motivational factor	Questions	Strongly disagree	Disagree	Neutral	Agree	Strongly agree
Community recognition	Do you feel that the communities you serve at the health posts genuinely need your services?	5 (1.6%)	6 (1.9%)	31 (9.9%)	66 (21%)	206 (65.6%)
	Do you believe that the changes in the community’s health status reflect an appreciation for your work?	4 (1.3%)	4 (1.3%)	37 (11.8%)	62 (19.7%)	207 (65.9)
Career development	Is there an established system in the Health Office for supporting the career development of Health Extension Workers?	49 (15.6%)	50 (15.9%)	80 (25.5%)	52 (16.6%)	83 (26.4%)
	Do you think career development opportunities enhance (motivate) the performance of Health Extension Workers?	10 (3.2%)	25 (8%)	50 (15.9%)	58 (18.5%)	171 (54.5%)
Supervision	Do you receive regular and constructive feedback from your immediate supervisor?	10 (3.2%)	30 (9.6%)	105 (33.4%)	66 (21%)	103 (32.8%)
	Do you believe that the feedback you receive from your supervisor motivates you to improve your performance?	9 (2.9%)	30 (9.6%)	61 (19.4%)	73 (23.2%)	141 (44.9%)
Training	Do you believe your job requires regular on-the-job or off-the-job training?	2 (0.6%)	7 (2.2%)	61 (19.4%)	72 (22.9%)	172 (54.8%)
	Do you agree your job requires a variety of knowledge and skills sharing to co-workers?	0 (0%)	6 (1.9%)	35 (11.1%)	66 (21%)	207 (65.9%)
Incentives and decision making	Are you satisfied with the current opportunities for promotions, transfers, and educational or training advancements in your work?	15 (4.8%)	29 (9.2%)	83 (26.4%)	57 (18.2%)	130 (41.4%)
	Do you have the autonomy to make decisions and perform your work independently?	8 (2.5%)	10 (3.2%)	58 (18.5%)	65 (20.7%)	173 (55.1%)

### Association between socio-demographic variables and motivation

The results of the binary logistic regression analysis examining the relationship between selected independent variables and the level of motivation among the HEWs are summarized in Table 3. Among the socio-demographic variables examined, marital status and years of service were significantly associated with motivation. HEWs who were married, divorced, or widowed demonstrated significantly higher odds of being motivated than their single counterparts. For instance, married HEWs had an AOR of 222.93 (95% CI: 12.92–3847.77; *p* < 0.05) (see Table 3). Similarly, years of service showed a positive and significant association with motivation: HEWs with 1–5 years of service had an AOR of 78.17 (95% CI: 1.54–3979.21; *p* < 0.05), 11–15 years of service had an AOR of 4.79 (95% CI, 1.25–18.43; *p* < 0.05), and more than 15 years of service had an AOR of 5.79 (95% CI, 1.57–21.34; *p* < 0.05). In contrast, age groups, educational level, and religious affiliation did not demonstrate significant associations with motivation. While these results underscore the potential influence of professional tenure and marital stability on HEWs motivation, the wide CIs suggest that these results need to be interpreted with caution.

**Table 3:** Binary logistic regression analysis of motivation level in relation to independent variables among HEWs.

Variables	Characteristics	Motivation		Crude OR (95% CI)	Adjusted OR (95% CI)
		No	Yes		
Age	18–27	46	108		
	28–37	8	26	0.58 (0.33, 1.02)	1.08 (0.17, 6.97)
	>38	25	101	0.80 (0.33, 1.99)	0.65 (0.12, 3.58)
Marital status	Single				
	Married	16	16	25.50 (3.79, 171.58)	222.93 (12.92, 3847.77)*
	Divorced	59	185	9.41 (1.85, 47.86)	25.91 (2.67, 251.88)*
	Widowed	4	34	4.20 (0.70, 25.26)	27.25 (2.15, 345.42)*
Religion	Muslim				
	Orthodox	1	2	1.33 (0.65, 2.73)	0.24 (0.00, 57.96)
	Protestant	67	188		0.33 (0.00, 85.91)
	Catholic	11	45	0.71 (0.06, 7.99)	0.00
Education	Grade 10–12				
	Certificate	57	170	5.06 (0.78, 32.98)	1.85 (0.01, 363.85)
	Diploma	3	9	4.47 (0.73, 27.45)	1.11 (0.06, 19.12)
	Degree	16	54	4.50 (0.49, 41.25)	0.65 (0.05, 9.19)
Service year	<1 year				
	1–5	27	56	0.53 (0.11, 2.63)	78.17 (1.54, 3979.21)*
	6–10	20	45	0.32 (0.07, 1.52)	1.66 (0.30, 9.32)
	11–15	15	52	0.71 (0.14, 3.47)	4.79 (1.25, 18.43)*
	>15 years	17	82	0.35 (0.07, 1.68)	5.79 (1.57, 21.34)*

Statistically significant (*p* < 0.05) are indicated with an asterisk.

The binary logistic regression analysis also assessed the association between selected organizational factors and the level of motivation among HEWs (Table 4). Neither walking time from residential areas to health posts nor gross monthly salary demonstrated significant association with motivation. Across all walking time categories, the AORs ranged from 0.91 to 1.74, with wide CIs and *p*-values > 0.05 (Table 4). Similarly, differences in gross monthly salary did not significantly influence HEW motivation. In contrast, the existence of performance related incentives significantly increased the odds of being motivated (AOR = 2.463; 1.499–4.048; C: 95%; *p* < 0.05). In addition, knowledge and skill sharing with co-workers (AOR: 3.52; 1.62–7.63; CI:95%; *p* < 0.05) and decision-making autonomy (AOR = 3.114; 1.677–5.783; CI: 95%; *p* < 0.05) were both positively associated with higher level of motivation. Conversely, inadequate implementation of the career development system was negatively associated with motivation (AOR = 0.510; 0.307–0.848; CL: 95%; *p* < 0.05), as were regular supervision and feedback (AOR = 0.503; 0.288–0.878; CI: 95%; *p* < 0.05) and access to on-the-job or off-the-job training (AOR: 0.44; 0.21–0.92; CI:95%; *p* < 0.05).

**Table 4:** Binary logistic regression analysis of the association between organizational, contextual, and motivational factors and motivation among HEWs.

Variables	Characteristics	Motivation		Crude OR (95% CI)	Adjusted OR (95% CI)
		No	Yes		
Distance from Woreda town	<1 Hour	12	25		
	1–2	16	35	1.75 (0.75, 4.09)	0.91 (0.21, 3.87)
	2–3	15	47	1.65 (0.68, 3.99)	1.62 (0.45, 5.89)
	3–4	16	55	1.05 (0.42, 2.60)	1.74 (0.48, 6.31)
	>4 Hours	20	73	1.50 (0.61, 3.70)	0.93 (0.21, 4.02)
Gross salary	<1,000 Birr				
	1,001–2000	26	64	0.41 (0.05, 3.58)	0.25 (0.01, 6.61)
	2001–3,000	53	171	0.53 (0.06, 4.49)	0.48 (0.02, 11.77)
	3,001–4,000				
	>4,001 Birr				
Recognition	Do you feel that members of the community recognize and value your work at the Health Post?	79	235	1.84 (1.39, 2.45)	0.83 (0.43, 1.61)
	Do you believe that improvements in the health status of the communities reflect appreciation for your work?	79	235	2.07 (1.54, 2.79)	1.25 (0.73, 2.13)
Career development	Is there a system in the Health Office that provides career development opportunities for HEWs?	79	235	1.01 (0.84, 1.21)	0.51 (0.31, 0.85)*
	Do you believe career development opportunities count as a motivational factor for HEWs?	79	235	1.83 (1.46, 2.29)	1.51 (0.98, 2.31)
Supervision	Do you receive regular supervision and feedback from your immediate supervisor?	79	235	1.16 (0.92, 1.45)	0.50 (0.29, 0.88)*
	Do you believe regular supervision and feedback from your supervisor encourages you to perform well?	79	235	1.66 (1.33, 2.08)	1.18 (0.65, 2.15)
Incentives	Are you satisfied with the performance related incentive opportunities (promotion, transfer, educational/training) for HEWs?	79	235	2.03 (1.61, 2.56)	2.46 (1.50, 4.05)*
Training	Do you believe your job requires regular on-the-job or off-the-job training?	79	235	2.05 (1.54, 2.72)	0.44 (0.21, 0.92)*
	Do you agree your job requires a variety of knowledge and skill transfer among co-workers?	79	235	3.32 (2.34, 4.72)	3.52 (1.62, 7.63)*
Decision making autonomy	Can you do your work the way you want independently?	79	235	2.72 (2.05, 3.61)	3.11 (1.68, 5.78)*

Statistically significant (*p* < 0.05) are indicated with an asterisk.

## Discussion

In this study, we assessed the socio-demographic characteristics and institutional determinants of motivation among HEWs draw from six Woredas and one town administration in East Hararge Zone, Ethiopia. The findings contribute valuable, context-specific insights into the drivers of motivation among frontline health workers operating in resource-constrained settings. These insights are not only relevant for strengthening health system performance and workforce sustainability in community-based primary healthcare delivery locally but also hold relevant for similar under-resourced settings elsewhere.

The overall motivation level of HEWs reported in this study (74.8%) appears higher than figures reported in previous studies in other regions of Ethiopia and in comparable low-resource settings elsewhere. For instance, lower motivation levels have been observed among primary health care workers in West Hararge (51.8%) (13), Hadiya (52.7%) (25), and Sidama (36.6%) (21) Zones in Ethiopia. In the Southern Nations, Nationalities, and Peoples’ Region (SNNPR), 61% of healthcare professionals rated their motivation as “very good” or “excellent” (24). Elsewhere, a study in Uganda found that only 37% of physicians working in hospitals reported being satisfied with their jobs (24). These inter-regional and cross-country differences in motivation are commonly attributed to a range of intrinsic and extrinsic factors, including years of experience, level of community engagement, health system infrastructure, supervisory support, and availability of career development opportunities (18, 26, 27).

Comparable challenges have been documented across other low- and middle-income countries (LMICs). In Sierra Leone, rural health workers reported motivation related challenges stemming from multiple factors, including difficult terrain, poor working conditions, limited access to training, and long working hours (28). In Malawi, lack of human resource management practices and inadequate continuous education and career progression contributed to demotivation among frontline health care workers (29). A systematic review by Willis-Shattuck et al. (5), spanning multiple LMICs, consistently identified lack of recognition, career stagnation, and inadequate compensation as key demotivating factors to health worker motivation. Nonetheless, the variability in findings across regions and countries underscores the context-dependent nature of health worker motivation, while also highlighting the widespread systematic challenges faced by under-resourced health systems.

The socio-demographic analysis revealed that the majority of HEWs in East Hararge Zone were young women, predominantly aged between 28 and 37 years, and married, a profile consistent with national patterns reported among HEWs across Ethiopia (30). The exclusively female composition of the sample aligns with the intentional design of Ethiopia’s Health Extension Program (HEP), which strategically prioritizes the recruitment of women to improve maternal and child health outcomes and bring about greater trust in community-based service delivery (16).

Marital status emerged as one of the significant predictors of motivation, with married HEWs, for instant, demonstrating higher levels of motivation compared to their single counterparts. This association may reflect the social, emotional, and logistical support provided by spouses (and other families members), which can help reduce occupational stress and promote greater work engagement and resilience. Similar findings have been reported in other Ethiopian studies, highlighting the influence of personal life circumstances in shaping professional satisfaction and retention among frontline health workers (31).

Years of service also showed a strong positive association with motivation. HEWs with longer tenure may benefit from cumulative access to training opportunities, improved competence, and greater trust and recognition from the communities they serve. These factors align with the Herzberg’s theory, which identifies achievement and recognition as key intrinsic motivators. These results are consistent with broader literature emphasizing the importance of experience and career progression in sustaining motivation in community health systems (5, 28, 29).

Although walking time between residential town and assigned health posts was not significantly associated with motivation in our result, the practical implications of long daily commuting warrant attention. Over two-thirds (68.4%) of HEWs reported walking between 1 to 4 hours daily to reach their health posts, while ca. 20% commuting over 4 hours each day. The extended commutes are not just inconvenient, they contribute to physical exhaustion, reduce time for personal and family responsibilities, and may ultimately undermine long-term job satisfaction and retention. These concerns are echoed in studies from rural Kenya and Uganda, where inadequate transport support and long commuting times negatively impacted health workers motivation and retention (32, 33). In Ethiopia, the lack of a transportation subsidy system compounds this burden, as commuting costs would be borne entirely by the HEWs, further stretching their already modest salaries. In East Hararge Zone, this challenge is compounded by the Zones dispersed settlements, rugged terrain, and underdeveloped transportation infrastructure, making daily commuting by foot physically demanding and unsustainable over time.

Institutional factors were also significantly and positively associated with higher levels of motivation among HEWs. Consistent with Self-Determination Theory, decision-making autonomy was strongly associated with higher motivation level in our study. This aligns with reports from Kenya and Nepal, where greater health workers autonomy has been positively linked to job satisfaction and a stronger sense of task ownership (31, 34–36). Furthermore, access to incentive opportunities and knowledge and skill sharing among co-workers were significantly associated with higher level of motivation, consistent with studies from other regions in Ethiopia (25), and broader literature emphasizing the importance of institutional support, recognition, and peer collaboration in sustaining workforce motivation (5).

While institutional support, including inadequate implementation of the career development system, regular supervision feedback, and training opportunities are typically expected to increase motivation of the HEWs, we found significant negative associations between these factors and motivation. These paradoxical observations could be due to experiences and perceptions of these mechanisms among HEWs as opposed assuming their mere existence guarantees motivational benefit.

Although formal career development systems are designed to increase job satisfaction and improve long-term retention (5), their success is contingent upon their transparency, perceived equity, and actual implementation. In low-resource settings, these systems often exist only on paper, less operationalized in practice. They have a few or no promotion options, contain vague or inconsistently applied standards of performance evaluation, and are often politically influenced. These challenges can lead to frustration and unfulfilled expectations among health workers, which in turn may contribute to staff demotivation (21, 37). Likewise, while effective supervision and feedback are important sources of support and development, their motivational impact depends on how well they are implemented. In practice, supervision and feedback often follow a top-down, inspection-oriented approach emphasizing fault finding instead of supportive and constructive approach (37). Such approaches may reduce autonomy and increase anxiety among health workers, rather than creating a positive environment for growth and engagement. Access to on-the-job or off-the-job training also showed a negative association with motivation. While access to training is widely recognized as an important factor for motivation, it may lose its value if it is not done well, repetitive, not aligned with the job at hand, or fails to lead to tangible outcomes (such as certification, new responsibilities, or incentives). In such contexts, training risks being viewed as an obligation rather than a pathway to growth and empowerment.

Finally, our findings reaffirm the relevance of the three fundamental theories of motivation mentioned in the introduction and demonstrate that they retain conceptual relevance and explanatory power in resource-constrained settings, supporting the view that motivational drivers are grounded in universal human psychological needs. From Maslow’s perspective (2), basic needs such as physical safety, job security, and manageable commuting conditions represent foundational motivators that must be met before higher-level needs can meaningfully influence behavior. In our study, the absence of transport subsidies, extended commuting hours, and lack of structural support likely impacted these basic needs. Once these are sufficiently addressed, higher-order motivational drivers such as recognition, opportunities for advancement, decision-making autonomy, and supportive collegial interaction that are central to professional growth and self-actualization will become more pertinent in sustaining motivation. Herzberg’s theory (3) offers a useful lens to distinguish between hygiene factors (e.g., salary, job security, supervision, and working conditions), which are essential to prevent dissatisfaction, and true motivators (e.g., career development opportunities, recognition, and participation in decision-making), which actively enhance job satisfaction and performance. Interestingly, some hygiene factors in our study (e.g., supervisory feedback and training) were negatively associated with motivation, most likely due to their punitive or poorly managed implementation, underscoring the importance of quality and perception over mere presence. The identification of decision-making autonomy and collaborative knowledge and skill-sharing as significant positive correlates of motivation is also consistent with Self-Determination Theory (4). This framework emphasizes that intrinsic motivation is driven by the fulfilment of three core psychological needs: autonomy (having control over one’s work), competence (feeling effective in one’s role), and relatedness (feeling connected to others). Our findings suggest that when HEWs are empowered to make decisions and share expertise with peers, they experience a stronger sense of ownership, capability, and community. These factors are especially critical for sustaining motivation in decentralized and under-resourced health systems.

## Conclusion

The findings of this study offer valuable insights into the multiple drivers of motivation among HEWs in East Hararge Zone, Ethiopia. Addressing the motivational challenges identified requires a careful, context-specific approach that considers socio-demographic and institutional dimensions. Strengthening performance related incentives, implementing decision making autonomy, and realizing collegial knowledge and skill sharing scheme can help mitigate career stagnation and reinforce motivation. Institutional factors found negatively associated with motivation warrant further study, not necessarily due to their existence per se, but rather due to issues surrounding their implementation, perceived quality and contextual relevance. Poor implemented career development schemes, lack of transparency, political influence, and top-down supervisory approaches may erode trust and contribute to a negative working environment, eventually undermining motivation and retention. To ensure long-term sustainability and effectiveness of the Health Extension Program and the broader primary healthcare system in Ethiopia, these institutional reforms must be embedded in policy and implemented diligently. Furthermore, recognizing and responding to regional variability in motivational determinants is essential for designing adaptive, context-specific strategies that strengthen health workforce performance and community health outcomes in diverse settings.

## Data Availability

The raw data supporting the conclusions of this article will be made available by the authors, without undue reservation.
